# Multi-Omics Analysis to Generate Hypotheses for Mild Health Problems in Monkeys

**DOI:** 10.3390/metabo11100701

**Published:** 2021-10-13

**Authors:** Fumie Hamano, Suzumi M. Tokuoka, Megumi Ishibashi, Yasuto Yokoi, Dieter M. Tourlousse, Yoshihiro Kita, Yuji Sekiguchi, Hiroyuki Yasui, Takao Shimizu, Yoshiya Oda

**Affiliations:** 1 Department of Lipidomics, Graduate School of Medicine, The University of Tokyo, Hongo 7-3-1, Bunkyo-ku, Tokyo 113-8654, Japan; fhamano@lscore.m.u-tokyo.ac.jp (F.H.); stokuoka@m.u-tokyo.ac.jp (S.M.T.); kita@m.u-tokyo.ac.jp (Y.K.); tshimizu@ri.ncgm.go.jp (T.S.); 2 Thermo Fisher Scientific K. K., Moriya-cho 3-9, Kanagawa-ku, Yokohama-shi 221-0022, Japan; megumi.ishibashi@thermofisher.com; 3 Mitsui Knowledge Industry Co., Ltd., Atago Green Hills MORI Tower, Atago 2-5-1, Minato-ku, Tokyo 105-6215, Japan; yokoi-yasuto@mki.co.jp; 4 Biomedical Research Institute, Advanced Industrial Science and Technology (AIST), Central 6, Higashi 1-1-1, Tsukuba 305-8566, Japan; dieter.tourlousse@aist.go.jp (D.M.T.); y.sekiguchi@aist.go.jp (Y.S.); 5 Department of Analytical & Bioinorganic Chemistry, Division of Analytical and Physical Chemistry, Kyoto Pharmaceutical University, 5 Nakauchi-cho, Misasagi, Yamashina-ku, Kyoto 607-8414, Japan; yasui@mb.kyoto-phu.ac.jp; 6 Department of Lipid Signaling, National Center for Global Health and Medicine, Toyama 1-21-1, Shinjuku-ku, Tokyo 162-8655, Japan

**Keywords:** multi-omics, redox, selenium, saturated fatty acids, lipid mediators, multiple sample sources, microbiota, metabolomics, lipidomics, metallomics

## Abstract

Certain symptoms associated with mild sickness and lethargy have not been categorized as definitive diseases. Confirming such symptoms in captive monkeys (*Macaca fascicularis*, known as cynomolgus monkeys) can be difficult; however, it is possible to observe and analyze their feces. In this study, we investigated the relationship between stool state and various omics data by considering objective and quantitative values of stool water content as a phenotype for analysis. By examining the food intake of the monkeys and assessing their stool, urine, and plasma, we attempted to obtain a comprehensive understanding of the health status of individual monkeys and correlate it with the stool condition. Our metabolomics data strongly suggested that many lipid-related metabolites were correlated with the stool water content. The lipidomic analysis revealed the involvement of saturated and oxidized fatty acids, metallomics revealed the contribution of selenium (a bio-essential trace element), and intestinal microbiota analysis revealed the association of several bacterial species with the stool water content. Based on our results, we hypothesize that the redox imbalance causes minor health problems. However, it is not possible to make a definite conclusion using multi-omics alone, and other hypotheses could be proposed.

## 1. Introduction

Living a healthy life and reaching old age without agony and disease are common aspirations in human society. However, numerous diseases have to be overcome in old age. These include widely recognized diseases such as cancer, dementia, and infectious diseases, as well as diseases that cannot be diagnosed using conventional techniques despite the presence of clinically evident symptoms. The pre-symptomatic state of disease is increasingly attracting the attention of researchers and healthcare professionals. Various disease models have been used to elucidate the mechanisms underlying disease development and to develop diagnostic and therapeutic strategies. In vitro experiments using cell lines are relatively inexpensive and suitable for high-throughput screening; however, there is a large difference between the findings of in vitro experiments and phenomena observed in the body in vivo. In addition, although mice are used extensively as experimental models, there are significant differences in the biology and behavior of rodents and humans. For example, mice do not naturally develop aging-related diseases such as atherosclerosis and diabetes, partially due to their short lifespan [1,2]. Therefore, non-human primates (NHPs) could serve as effective experimental models, as they are close to humans in terms of evolutionary distance [3]. Furthermore, unlike research on humans, research on NHPs allows for complete control over housing, environment, diet, and behavior.

A survey investigating the experiences of veterinary clinicians working on diarrhea in primates in 1981 revealed the incidence of diarrhea in 13,385 monkeys to be 10.6% [4]. However, a 2018 study reported diarrhea in 3.6% to 31.6% of monkeys housed under captivity [5]. If these symptoms do not improve, they may lead to death or engender the need for euthanasia. Previous studies have repeatedly shown that both personality and psychological stressors can serve as predictors of gastrointestinal disease and chronic diarrhea [5]. In general, the risk of diarrhea is higher when one is nervous, introverted, and/or anxious. While certain stressful environments increase the risk of chronic diarrhea, the relative effects of such stressors are considered to be highly dependent on the animals’ personality [5]. In humans, diarrhea is one of the most common bowel diseases, affecting 8–20% of the general population globally [6]. In particular, irritable bowel syndrome (IBS), with diarrhea as a typical symptom, seems to be associated with high psychosocial stress and low quality of life and work productivity [7,8]. However, the mechanisms underlying the development of IBS are largely unknown. Chronic psychological stress is thought to play an important role in the onset and worsening of symptoms of functional bowel disease [8,9].

To study the complex biological processes in a comprehensive manner, an integrated approach involving omics analyses of different samples such as plasma/serum and urine, was undertaken to reveal the interrelationships between the biomolecules and their functions. This may facilitate a more comprehensive understanding of health and disease [10,11,12,13]. In multi-omics, a correlation is identified between a vast array of molecular profile information and the disease status. Therefore, it is important to evaluate the phenotypes of diseases as continuous and objective quantitative values. In the present study, stool water content was considered a phenotype [14,15], and multi-omics data pertaining to plasma, urine, feces, and food were obtained from monkeys housed in a controlled environment.

## 2. Results

### 2.1. Stool Water Content Analysis

The amount of water in each fecal sample is shown in Table 1. The values were obtained by weighing approximately 50 mg of stool, and the dry weight was obtained after drying the sample for 24 h in a dryer. The feces of five monkeys marked with an asterisk had high water content and were observed to be soft (only one of the animals (No. 20) had obvious diarrhea), suggesting that water content in the feces reflects the stool condition. The stool water content was considered a quantitative and an objective phenotype, and an association was investigated with the following omics data.

### 2.2. Metabolomics

**Figure 1:**
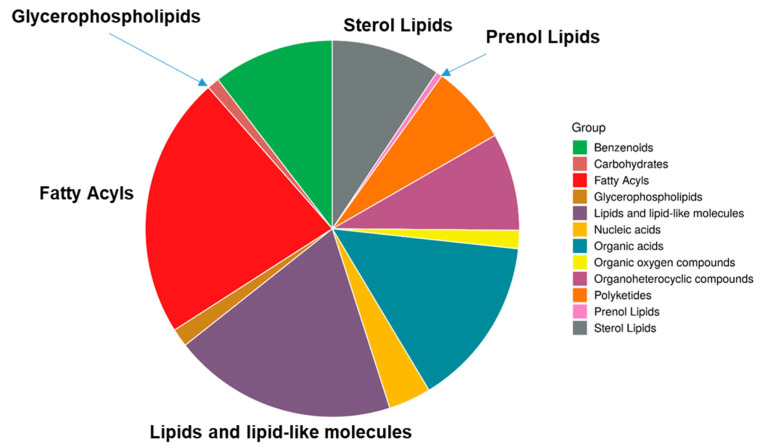
Classification of fecal metabolites that strongly correlate with stool water content.

Using untargeted metabolomics analyses, we detected 899 peaks in the plasma (Table S1), 2920 peaks in urine (Table S2), and 3125 peaks in feces (Table S3). As the number of monkeys in the present study was small (*n* = 20), we excluded peaks with missing values (below the detection limit). The number of peaks with significant correlations (*p* < 0.05) was as follows: 85 for plasma metabolites, 267 for urinary metabolites, and 1347 for fecal metabolites. Among them, the metabolites with particularly strong correlations (peaks with a strong correlation [±0.8]) with stool water content were examined using Pearson’s correlation coefficient. Such metabolites were not found in plasma or urine. However, 18 metabolites in stool had a positive correlation and 92 had a negative correlation with the stool water content. When the strength of the correlation was expanded to ±0.7, 392 peaks were correlated (along with three and two peaks in urine and plasma, respectively). We attempted to identify the metabolites using the “LC-MS Search” function (https://hmdb.ca/spectra/ms/search (accessed on 28 June 2021)) in the Human Metabolome Database (HMDB) with an accurate mass number. We speculated the names of 982 metabolites (HMDB ID numbers), including redundancies, such as structural isomers and stereoisomers (Table S4). As we did not use any standard metabolites for confirmation, it was difficult to specifically identify the metabolites, and some of the metabolites were considered misidentified. However, while misidentifications are likely to occur randomly, metabolites were correctly identified by annotating the characteristics of candidate metabolites to determine the metabolic pathways/networks most likely to be affected by these metabolites and the populations of metabolites with similar characteristics [16,17,18,19]. Therefore, we used this information to perform an analysis using the Enrichment Analysis function in MetaboAnalyst 5.0 (https://www.metaboanalyst.ca/ (accessed on 28 June 2021)). As shown in Figure 1, more than a half (53.4%) of the metabolites whose structures could be estimated by HMDB were lipid-related metabolites. Hence, we focused our subsequent analysis on lipids.

Liquid chromatography/mass spectrometry identification was performed using HMDB (https://hmdb.ca/spectra/ms/search (accessed on 28 June 2021)) by refining the precise molecular weight information to an error of 2 ppm. The obtained HMDB ID numbers were used in MetaboAnalyst 5.0’s Enrichment Analysis (https://www.metaboanalyst.ca/MetaboAnalyst/Secure/enrichment/ParamView.xhtml (accessed on 28 June 2021)). The super-class was selected from the chemical structures category to obtain a pie chart of the type of compound families correlated with stool water content.

### 2.3. Fatty Acid Analysis

**Figure 2:**
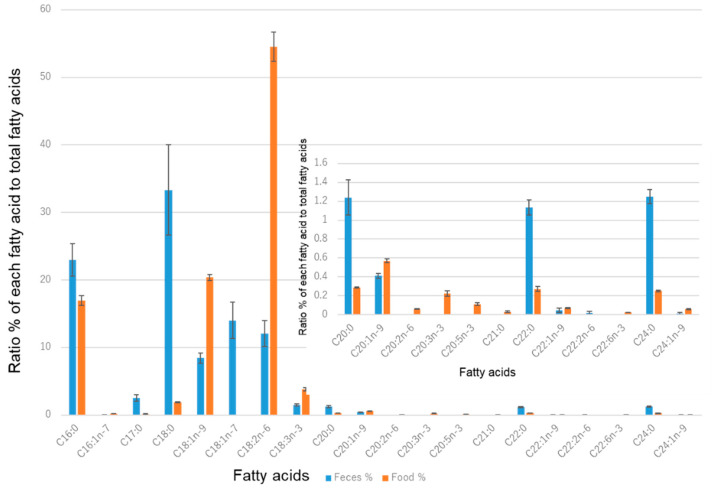
Comparison of fatty acid species in food and feces. The orange bars represent the fatty acids in the food (repeatedly twice), the blue bars the fatty acids in the feces (average of 20 animals, and each sample was measured repeatedly three times), the vertical axis shows the percentage when the total fatty acid content is 100%, and the horizontal axis shows the fatty acid species; the number following “C” indicates the length of the carbon chain, the number following the colon indicates the number of unsaturated bonds, and the number following “n-” indicates the position of the first unsaturated bond counting from the position of the carbon atom of the terminal methyl group. The graph embedded in Figure 2 is a magnified view of fatty acids with chains of more than 20 carbons. The error bars indicate the standard error.

To examine the lipids in more detail, the basic structural units of lipids and fatty acids were analyzed using gas chromatography (GC). Free fatty acids and fatty acids covalently bound to-cholesterol and -glycerol skeletons via ester bonds were hydrolyzed so that the total amount of fatty acids could be determined. The results are shown in Figure 2. The most common fatty acid in the feed (orange bars) was C18:2n-6 (54.5%), followed by C18:1n-9 (20.4%), and the two accounted for approximately 75% of the total fatty acid content. Most of the unsaturated fatty acids present in the diet were reduced to the saturated form in the feces, except for the C18 series, the levels of which increased in the feces.

**Figure 3:**
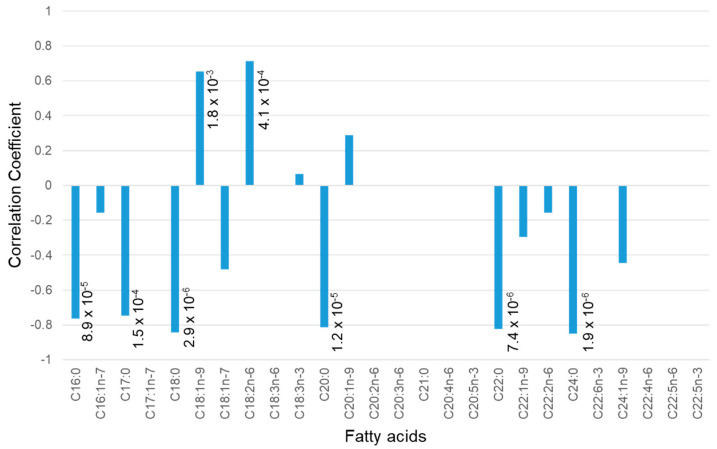
Correlation between water content and fatty acid content in feces. The correlation coefficient was calculated based on Pearson’s correlation coefficient and is shown on the vertical axis. The horizontal axis shows the type of fatty acid; the number following “C” indicates the length of the carbon chain, the number following the colon indicates the number of unsaturated bonds, and the number following “n-” indicates the position of the first unsaturated bond counting from the position of the carbon atom of the terminal methyl group. The fatty acids were measured by GC after derivatization. The numbers next to the bar graph are the *p*-values.

As shown in Figure 3, the correlation of fatty acids present in feces with stool water content was determined next. Two fatty acids, C18:2n-6 and C18:1n-9, positively correlated with the stool water content. These two fatty acids were abundant in the diet. Thus, in monkeys with defective intestinal absorption, the composition of the food may be strongly reflected in their feces. In other words, these two lipids were not metabolized completely and remained in the stool as food residue. Saturated fatty acids showed a strong negative correlation with stool water content (less than −0.8). The negative correlations between saturated fatty acids and stool water content may be due to the relatively low dry weight of wet stools.

However, as shown in Figure 3, some lipids were positively correlated, and even among lipids that were negatively correlated, large differences were observed in the strength of the correlation. We realized that the relationship between stool dry weight and stool lipids is not simple. Although the origin of the saturated fatty acids remains unknown, there was no significant correlation between plasma fatty acids and stool water content (Table S5). The levels of urinary fatty acids were not measured because the quantitative sensitivity of the assay was insufficient for the 200 µL urine obtained in this study.

### 2.4. Lipidomics

Untargeted lipidomic analyses were conducted to determine the source of saturated fatty acids in the stool. As only LipidSearch [20,21] was used for the analysis, and no verification using standard products was conducted, there were potential incorrect identifications. However, we decided to focus on lipid subclasses rather than the individual molecules identified.

**Figure 4:**
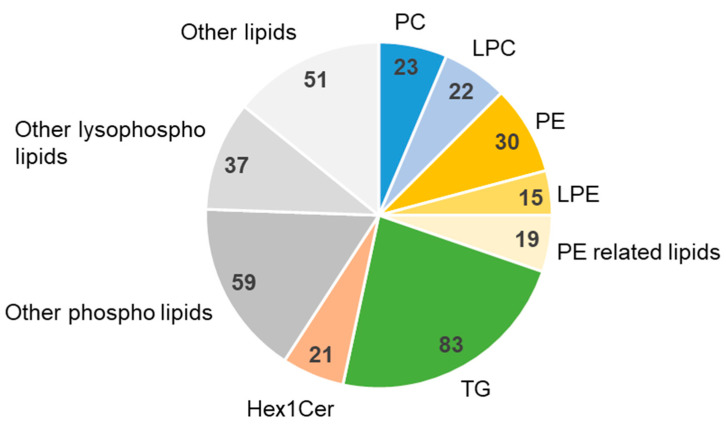
Lipidomics of monkey food. The numbers represent the number of lipids identified. PE-related lipids are LdMePE (5 lipids) and dMePE (14 lipids). PC, phosphatidylcholine; LPC, lysophosphatidylcholine; PE, phosphatidylethanolamine; LPE, lysophosphatidylethanolamine; TG, triglyceride; Hex1Cer, hexosylceramide.

We performed lipidomic analyses on monkey feed and identified 361 lipid components (Table S6), as shown in Figure 4. More molecular species belonged to TG than the other lipids, but the distribution (the number of lipid-species within one lipid class) of other lipids seemed to be relatively well-balanced. Subsequently, we conducted fecal lipidomic analysis, and 844 lipids were identified (Table S7).

**Figure 5:**
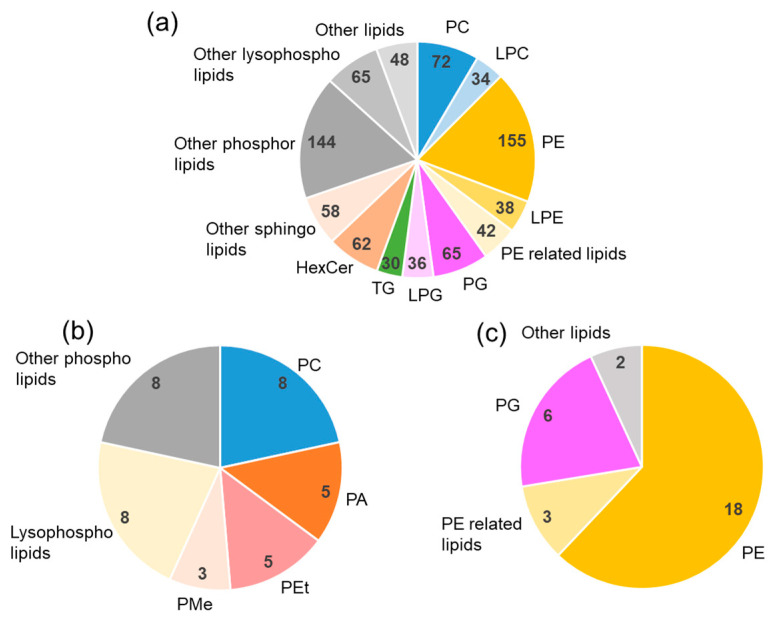
Fecal lipidomics. (**a**) Distribution (the number of lipid-species within one lipid class) of lipid components in the stool. (**b**) Lipids in stool with a significant positive correlation with the stool water content. (**c**) Lipids in stool with significant negative correlation with the stool water content. PC; phosphatidylcholine, LPC: lysophosphatidylcholine, PE, phosphatidylethanolamine; LPE, lysophosphatidylethanolamine; PG, phosphatidylglycerol; LPG, lysophosphatidylglycerol; TG, triglyceride; HexCer, hexosylceramide; PA, phosphatidic acid; Pet, phosphatidylethanol; PMe, phosphatidylmethanol.

Figure 5a shows the distribution (the number of lipid-species within one lipid class) of lipid components in the stool. The content of TG, which was abundant in the food, significantly decreased, and that of PE increased in the stool. The constituents in the stool are difficult to interpret because they are a mixture of undigested food residues and the molecules produced by the intestinal bacteria. It has been reported that PC is the most abundant phospholipid produced by mammalian cells [22,23]. In the present study, the number of phospholipids in the monkey diet was almost the same for PC/LPC and PE/LPE (Figure 4). However, in stool, PE/LPE was 1.5 times more than PC/LPC (Figure 5a). It has been reported that PE is more abundant in bacteria than PC [24,25]. The results suggest that the bacteria in the intestine produce various types of PE/LPE. Although we do not have clear explanations for the trends, fecal lipids with a positive correlation (*p* < 0.05) with stool water content are shown in Figure 5b. PA, PEt, and PMe were characteristic minor components in the stool. Figure 5c shows the lipids with a negative correlation. As the amount of water absorbed from the intestine decreases (amount of water in the stool increases), the amount of PE absorbed from the intestine may also decrease. However, if the decrease in absorption is not limited to PE, then the level of PE produced by the bacteria may reduce as the stool water content increases.

There were no obvious lipids in the stool that could explain the origin of the saturated fatty acids that inversely correlated with the water content as shown in Figure 3. Therefore, plasma lipidomic analysis was performed to identify 711 lipids, which were less than the molecular species identified in the fecal lipidomic analysis (Table S8).

**Figure 6:**
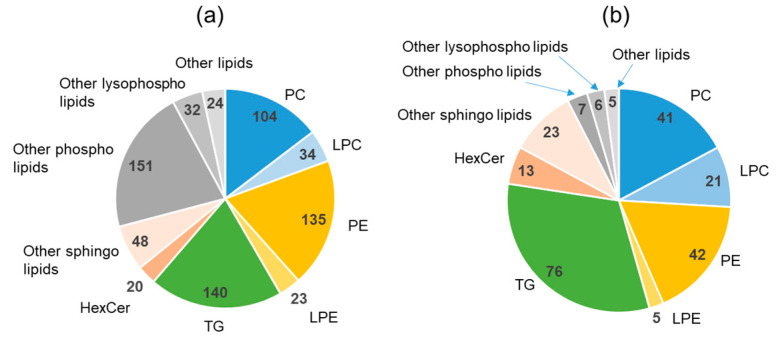
Plasma lipidomics. (**a**) Distribution (the number of lipid-species within one lipid class) of lipid components in the plasma. (**b**) Lipids in plasma with significant negative correlation with the stool water content. PC, phosphatidylcholine; LPC lysophosphatidylcholine; PE, phosphatidylethanolamine; LPE, lysophosphatidylethanolamine; TG, triglyceride; HexCer, hexosylceramide.

The distribution (the number of lipid-species within one lipid class) of plasma lipid species is shown in Figure 6a. The TG component abundant in the food was also abundant in the plasma, which was very different from the lipid component in the stool. In addition, the percentage of glycerophospholipids, including PC and PE, increased in the plasma compared with that in the food. Five plasma lipids (two each of PC and PE, and one of PG) positively correlated with the stool water content, although the correlation was not strong (0.5 < r < 0.6). However, there were 239 lipids with a negative correlation (*p* < 0.05), and their distribution (the number of lipid-species within one lipid class) is shown in Figure 6b.

Nineteen of the negatively correlated plasma lipids presented correlation coefficients of −0.7 or lower with the stool water content, which was considerably higher than that observed for lipids in the stool (Table S7). Of these, 10 were TGs, and the abundance of TGs was also characteristic, as shown in Figure 6b. LPC also tended to be abundant as a plasma lipid with a negative correlation, and four of them presented correlation coefficients of −0.7 or lower with the stool water content.

**Figure 7:**
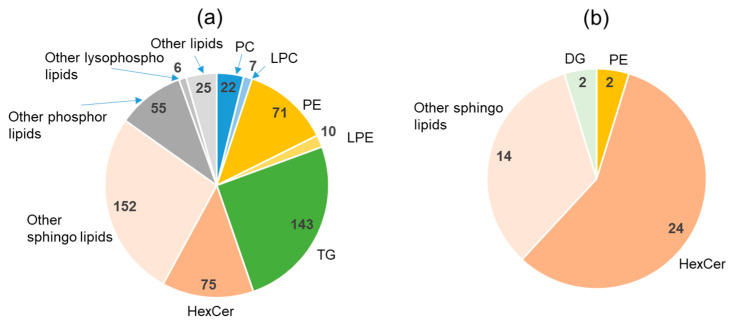
Urinary lipidomics. (**a**) Distribution (the number of lipid-species within one lipid class) of lipid components in the urine. (**b**) Lipids in urine with a significant positive correlation with the stool water content. PC, phosphatidylcholine; LPC: lysophosphatidylcholine; PE, phosphatidylethanolamine; LPE, lysophosphatidylethanolamine; TG, triglyceride; HexCer, hexosylceramide; DG, diglyceride.

Lipidomic analysis of urine samples led to the identification of 566 lipids in urine, which were fewer than the lipids found in stool and plasma, but more than those present in food (Table S9). As shown in Figure 7a, the relatively high number of TG species followed the same trend as in plasma, but a higher number of PEs than that of PCs was more pronounced in urine than in stool. The very high percentage of sphingolipids is probably a characteristic of urinary lipids. There were 42 urinary lipids that positively correlated with the stool water content (Figure 7b). All correlation coefficients with stool water content were less than 0.6 and were not as high as those of plasma lipids. However, it is noteworthy that the correlations were positive, unlike those of plasma and feces. Plasma circulates in the body, whereas stool is excreted and contains molecules of both bacterial and food origin. Only urine is considered as pure excretion from the body, and therefore it may be different from plasma and feces in terms of its interactions (in/out/circulation) with the body. Most of the urinary lipids with a positive correlation were sphingolipids. Although TG and PE were also common as urinary lipids, they did not correlate well with the stool water content. Both plasma TG and PE have been found to be inversely correlated with the stool water content. Only one type of lipid, PS, presented a negative correlation with the stool water content, and the correlation coefficient was −0.45 (not a strong correlation).

### 2.5. Lipid Mediator Analysis

In addition to general lipidomic analysis, we also measured the levels of fatty acid-derived lipid mediators and their related metabolites (FA metabolites) for functional analysis of the feces; 14 FA metabolites in the stool (Table S10) and 2 FA metabolites in plasma (Table S11) positively correlated with the stool water content (only oxidized lipids that were above the lower limit of quantification). Among them, six FA metabolites with relatively high correlation coefficients (0.6 or higher) are shown in Table 2. In addition, urinary FA metabolites, which were detected in 20 animals, did not correlate with the stool water content (Table S12).

The precursor of the oxidized fatty acids (excluding 13-HOTrE) was linoleic acid (LA, C18:2n-6). As shown in Figure 2, LA constitutes more than half of the fatty acids in monkey feed. It will be interesting to verify if these oxidized FAs decrease when the LA content in the diet is reduced. LA was also the precursor of two FA metabolites in plasma (9-KODE and 9-HpODE), which were positively correlated. These oxidized fatty acids in stool, particularly 13-HpODE, promote intestinal inflammation [26,27] and may affect the intestinal conditions and cause soft stools, even if they do not lead to specific diseases. However, due to oxidative stress, the production of 13-HpODE and other substances may have increased, causing intestine inflammation and poor food absorption. Two of the oxidized fatty acids in the plasma also correlated with the stool water content, suggesting that monkeys with soft stools may have increased oxidation in not only their stool but also their plasma.

### 2.6. Metallomic Analysis

**Figure 8:**
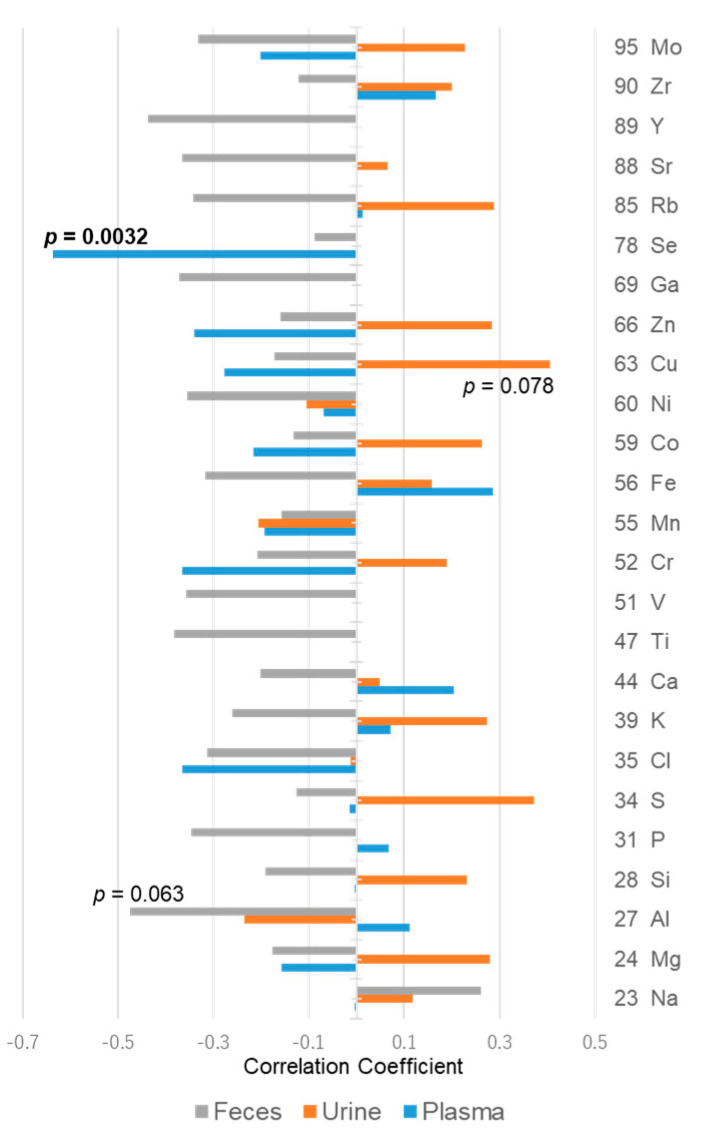
Correlation coefficients between trace elements in plasma, urine, or feces, and stool water content. The numbers next to the bar graph are the *p*-values. There were no significant differences (*p* > 0.05) except for the two mentioned on the graphs. Gray bars: feces samples; orange bars: urine samples; and blue bars: plasma samples.

In general, the concentration of ions in the gastrointestinal tract changes the osmotic pressure in the lumen, which in turn affects the stool water content. Therefore, the trace elements, including metallic elements that constitute inorganic substances in feces, plasma, and urine, were also measured in this study. The correlations with stool water content are shown in Figure 8 and Table S13.

The only element in plasma with correlation coefficients higher than 0.5 or lower than −0.5 was selenium (Se), which showed a negative correlation with the stool water content (−0.637, *p* = 0.0025); however, no such correlation was found in stool or urine. Although Se is an essential element and exhibits antioxidant properties, it is incorporated into proteins as selenocysteine in vivo and is predominantly found as selenoprotein P in the plasma [28,29]. Glutathione peroxidase (GPx), one of the selenoproteins, plays an important role in the reduction of lipid peroxides. GPX4 is an essential selenoprotein in mammals and regulates lipid peroxidation by utilizing selenocysteine [30]. The decrease in Se in the plasma may increase the content of oxidized fatty acids. However, as fatty acids other than 9-HpODE and 9-KODE did not change notably in the plasma, some other mechanism may also be involved. Metallomic analysis of trace elements in the food revealed that all the elements shown in Figure 8 were present in the food (Table S14). However, recent studies have shown that Se, an essential trace element for living organisms, is stored in intestinal bacteria and is utilized by the host as and when needed [31]. Therefore, we then investigated the intestinal bacteria from feces.

### 2.7. Microbiota Analysis

**Figure 9:**
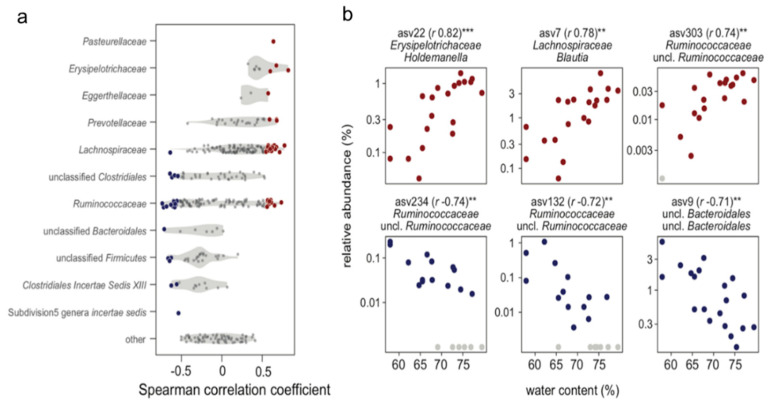
Fecal microbial diversity and their relationship with fecal water content. (**a**) Summary of Spearman’s correlation coefficients reflecting the relationship between ASV abundance and water content. Violin plots show the distribution of correlation coefficients for all ASVs analyzed and grouped according to taxonomic affiliation (*y*-axis). Non-significant correlation coefficients (adjusted *p*-value threshold of 0.1) are shown as dark gray circles, and significant negative and positive correlations are plotted as blue and red circles, respectively. (**b**) Scatter plots of ASV abundance and water content for ASVs with the most significant positive (top) and negative (bottom) correlations (*** adjusted *p*-value of <0.01 and ** adjusted *p*-value of < 0.05). Graphics for all ASVs with significant correlations are shown in Supplementary Figures S1 and S2. Note that ASVs with zero abundances in the rarified ASV tables are shown as light gray symbols, plotted at a pseudo-abundance of 0.001%.

Based on microbiota profiling by 16S rRNA gene amplicon sequencing, we further assessed the correlation between the abundance of amplicon sequence variants (ASVs) and the stool water content. For this analysis, we considered ASVs that were detected in the feces of at least half the monkeys (based on the mean of rarified ASV tables of duplicate measurements per monkey, see the Methods). This subset of ASVs (*n* = 405) accounted for 91.7% ± 2.2% (mean and standard deviation) of total abundances across samples or monkeys. Using Spearman’s correlations to identify monotonic associations, we recovered 60 ASVs that were significantly (adjusted *p*-value of <0.1) correlated with the stool water content (Figure 9a and Supplementary Figures S1 and S2).

Within the family Lachnospiraceae, the abundance of ASVs belonging to multiple genera, including *Blautia*, *Roseburia*, and *Dorea*, positively correlated with the stool water content and increased up to an order of magnitude in abundance (as a percentage of the total community) across the range of the stool water content evaluated (Figure 9b). Other ASVs that positively correlated with the water content included ASVs from members of the genera *Holdemanella* and *Faecalibacillus* (family Erysipelotrichaceae), and *Prevotella* (family Prevotellaceae). For the family Ruminococcaceae, correlation coefficients had both significant positive and negative associations with the water content. Many ASVs that negatively correlated with the water content could be identified only at higher taxonomic levels. Based on the ASV table collated at the family level (that is, by summing ASV abundances within families), Lachnospiraceae significantly positively correlated with the stool water content, which is consistent with the positive correlations for a considerable number of ASVs within this family, whereas Ruminococcaceae negatively associated with the stool water content, although the correlation was not significant (Supplementary Figures S1 and S2).

In line with mounting evidence that the families Lachnospiraceae and Ruminococcaceae play an important and potentially dual (beneficial and detrimental) role in the development of intra- and extra-intestinal diseases [32,33,34], correlational analysis also suggested an association between both families and mild health conditions in our monkeys. Although the genera *Dorea* and *Blautia* within the family Lachnospiraceae are generally considered part of a healthy gut microflora, increased abundance of *Dorea* has been reported in Japanese individuals with diarrhea [35], whereas enrichment of Blautia has been shown in the stool of individuals with self-reported bowel symptoms [36]. Furthermore, the family Erysipelotrichaceae has been linked to inflammation-related gastrointestinal diseases [37], and Nakajima et al. [38] reported a positive correlation between the genus *Holdemanella* and diarrhea in patients with type 2 diabetes mellitus. In foals, Schoster et al. [39] reported underrepresentation of the families Ruminococcaceae and Lachnospiraceae in fecal samples of subjects with diarrhea. Although we observed a significant decrease in some ASVs within the family Ruminococcaceae with an increase in stool water content, other Ruminococcaceae-related ASVs exhibited an opposite trend.

Overall, fecal microbiota analyses results suggested several microbial signatures that may be indicative of intestinal dysbiosis in monkeys with elevated stool water content. However, the possible role(s) of the bacteria in modulating host physiology, especially redox balance, remains to be elucidated.

## 3. Discussion

One of the shortcomings of using multi-omics approaches to analyze individual subjects (humans and animals) is the handling of phenotypes. Especially in the case of non-defined diseases, there are only qualitative indices, and they do not fit well with quantitative omics data. In the present study, we obtained continuous quantitative data by using water content in stool as a phenotype for reflecting the health status of monkeys, in monkeys housed for a long time under similar conditions and controlled for infections. The observation that the amount of saturated fatty acids in stool decreased as the stool became softer (as the stool water content increased) was interesting because it has also been reported that an increase in saturated fatty acid levels in the lumen of the colon improves gastrointestinal motility [40]. Fatty acids other than free fatty acids are often stored as TG [41]. The molecular species that were correlated strongly (inverse correlation) with the stool water content were saturated fatty acids in stool, followed by TG in plasma. It has been reported that the plasma TG levels and oxidative status in plasma are inversely correlated [42,43]. Metal ion analysis by inductively coupled plasma-mass spectrometry (ICP-MS) revealed a deficiency of Se in plasma, and plasma Se was also inversely correlated with stool water content. As Se has antioxidant properties, plasma may be inclined to the oxidative status. Selenoprotein P is an enzyme involved in the metabolic pathway of PE [44]. Similar to Se, many plasma PEs (major phospholipids in bacteria) are inversely correlated with stool water content, and PEs are also more predominant in the stool. Furthermore, phospholipase D is activated by reactive oxygen species [45,46], and it is known to produce PA and Pet [47,48]. Therefore, in soft stools, the PA and PEt levels may have increased due to the components of the stool tending to the oxidative status. In diabetic nephropathy, oxidative stress increases sphingolipid levels [49], and sphingolipids reportedly regulate redox homeostasis in chronic kidney disease [50]. Although our study was not related to kidney disease, urinary sphingolipid levels increased with soft stools, which may reflect oxidative stress, and which may be consistent with other observations. Several studies have reported that oxidative stress is involved in inflammation and affects gut health [51,52].

Some phenomena cannot be directly explained by the redox state. Conflicting results have been reported by disease lipidomic studies performed on human subjects. For example, it has been reported that the plasma LPC levels increase under some inflammatory conditions, and decrease under other inflammatory conditions [53]. However, the mechanisms of action of some lipids are complex, and the interpretation of the results is not simple due to the complex interplay of numerous factors. In this study, intestinal bacteria were associated with soft stools, but the detailed involvement of intestinal bacteria in the mechanisms of action remains unknown. Based on the results of this study, we hypothesized that a disturbance in redox balance may cause mild health problems. However, this is not a comprehensive multi-omics study, and there is a lack of data to test the hypothesis, and other hypotheses can be developed. In addition, the reasons for soft stools may be multifactorial and may differ among individual monkeys. It may be difficult to develop a hypothesis with only 20 individuals. However, by systematically examining the food that is ingested, the feces and urine that are excreted, and the plasma of an individual, it is possible to evaluate the changes occurring in the body. In this study we only collected samples at one time point, but by examining the changes over time, a hypothesis about the changes occurring in the body can be developed.

## 4. Materials and Methods

### 4.1. Reagents

Phospholipid mixtures were obtained from Avanti Polar Lipids, Inc. (Alabaster, AL, USA). Lipid mediator standards and deuterium-labeled compounds used as internal standards were obtained from Cayman Chemical Company (Ann Arbor, MI, USA). Trace element standard solutions, special grade reagents of ammonium bicarbonate, sodium chloride, diethyl ether, and chloroform, nitric acid and perchloric acid of toxic metal determination grade, hydrogen peroxide for atomic absorption spectrometry, and high-performance liquid chromatography-grade solvents (methanol, isopropanol, and acetonitrile) were purchased from Wako Pure Chemical Co. (Osaka, Japan) and from Kanto Chemical Co., Inc. (Tokyo, Japan, for untargeted lipidomics). Deionized water was produced using a Millipore-Q water system (Millipore, Bedford, MA, USA).

### 4.2. Samples

Plasma, urine, and stool samples from 20 male monkeys (Macaca fascicularis, known as cynomolgus monkeys), aged 3 years 10 months to 6 years 5 months, housed for more than eight months under the same conditions, were obtained from LSI Medience Co. (Tokyo, Japan) after a review by the Animal Experimentation Committee, and approval by the Director of the Testing and Research Center in LSI Medience Co. (Approval No. 2018-1071). All methods were carried out in accordance with relevant guidelines and regulations. The monkeys were housed with the lights turned on from 7:00 to 19:00 h and turned off at night. The cages were cleaned once a day. The monkeys were fed 100 g of solid feed (CLEA Old World Monkey Diet CMK-2; CLEA Japan Inc., Tokyo, Japan) once a day. There was no solid leftover food inside the cages; however, it was possible that the food was crushed or thrown outside the cage and the food given was not consumed completely. Filtered (5-µm filter) and UV-irradiated tap water was provided as drinking water to the monkeys. Monkeys have a habit of urinating when the lights are turned on in the morning; therefore, urine was collected early in the morning by placing a urine-collection tray in the home cage and lighting up the animal room. Regarding stool collection, as we did not know when the monkeys would defecate, a tray for feces was placed in the home cage, and the animal caretakers collected the stool into a plastic tube as soon as they found one; this may have happened on a different date than the date of urine collection. On the same day as urine collection, approximately 5 mL blood was drawn from a monkey and collected in a tube with EDTA-2K and centrifuged (1750× *g*, 10 min, 4 °C) to obtain approximately 2 mL plasma. All samples were frozen and stored in a −80 °C freezer at LSI Medience immediately after collection and placed on dry ice during transport.

### 4.3. Sample Preparation

To carry out targeted lipidomic/fatty acid analysis by gas chromatography with flame ionization detection (GC-FID), 50 µL of plasma samples and 20 mg wet weight of feces and feed (the feces and feed were dissolved while dipped in a sonication bath, and the feed was fully pulverized using a stainless-steel crusher) were derivatized using the Fatty Acid Methylation Kit (Nacalai Tesque, Tokyo) with C23:0 fatty acid solution (n-tricosanoic acid; Sigma-Aldrich, St. Louis, MO, USA) as the internal standard.

As described in the 2.2 Metabolomics section, metabolomics was performed first. Methanol extraction is commonly used in metabolomics. As shown in Figure 1, there were many lipid-related molecules. Therefore, methanol extractions were used for lipidomics in order to provide consistency in the results.

For untargeted lipidomics by LC/MS, 20 µL plasma was combined with 180 µL methanol, vortexed, and centrifuged to collect the supernatant. The supernatant was further diluted three times with methanol for the analysis. Since the urine sample was dilute, the concentration was needed. Urine lipids were recovered from the organic layer of 300 µL urine using the Bligh and Dyer method, followed by evaporation of the organic solvent, and resuspension of the residuals in 60 µL methanol for measurement (the upper layer was used for metabolomics). Approximately 20 mg feces was weighed (wet weight) and mixed with 20 µL water to avoid aggregation, and placed on ice. After vortexing, 180 µL methanol was added, and the mixture was stirred in an ultrasonic bath with ice cooling. After centrifugation, the supernatant was diluted four times with methanol and used to measure phospholipids. Twenty milligrams of feed was ground and extracted with 500 µL methanol. After centrifugation, the supernatant was collected and diluted with methanol to 5 mg/mL.

For targeted lipidomics by LC/MS, solid-phase extraction (SPE) was performed as described previously with some modifications [54]. Briefly, 200 µL plasma was combined with 0.8 mL methanol and mixed with internal standards (18 heavy water-labeled components) and centrifuged to collect the supernatant. The supernatant was then purified using an Oasis HLB cartridge (10 mg; Waters, Milford, MA, USA) and eluted with 200 µL of 0.2% formic acid in methanol. The solvent was evaporated by rotary evaporator and resuspended in 50 µL methanol for measurement. Four hundred microliters of urine was centrifuged after mixing with 800 µL methanol and internal standards, and the supernatant was applied to the Oasis HLB cartridge, as mentioned above for plasma. The eluate was measured. Approximately 150 mg feces was measured as wet weight, and 300 µL water was added to the sample on ice and mixed well. Subsequently, 1.2 mL methanol and internal standards were added and mixed in an ultrasonic bath while chilled. The supernatant was purified using an Oasis HLB cartridge, as mentioned above for plasma samples, and eluted with 200 µL of 0.2% formic acid in methanol. The eluate was analyzed by LC/MS.

To carry out untargeted metabolomics for hydrophilic metabolites analysis by LC/MS, 560 µL methanol was added to 140 µL plasma. The sample was vortexed and centrifuged; then, 600 µL supernatant was concentrated in a rotary evaporator and resuspended in 100 µL methanol for measurement. For urine, the upper layer (aqueous phase) obtained in the Bligh and Dyer extraction with 300 μL urine was dried in a rotary evaporator and resuspended in 100 µL methanol for measurement. For feces, approximately 20 mg wet weight of feces was suspended in 20 µL water on ice, followed by the addition of 180 µL methanol; the sample was mixed in an ultrasonic bath while chilled. After centrifugation, the supernatant was diluted four times with methanol and measured. Twenty milligrams food was smashed, mixed well with 800 µL methanol, centrifuged to collect the supernatant, and then diluted 10-fold with methanol for measurement.

For trace element measurements, each sample (50 mg of feed samples, 50 μL each of plasma samples, 50 μL each of urine samples, and the entire amount of each feces sample) was placed in a 50-mL beaker and heated on hotplates to 150 °C. After 3 min, 2 mL of 60% (*v/v*) nitric acid (HNO_3_ for poisonous metal determination; Kanto Chemical, Tokyo, Japan) was added; 3 min later, 2 mL of 60% (*v/v*) perchloric acid (HClO_4_ for poisonous metal determination; Kishida Chemical, Osaka, Japan) added, and 3 min later, 2 mL of 30% (*v/v*) hydrogen peroxide (H_2_O_2_ for atomic absorption spectrochemical analysis; Kishida Chemical, Osaka, Japan) was added. This process was repeated three times, and if any brownish residue remained, the steps were repeated further until the sample was completely white. Thus, washing was continued until the residual material at the bottom of the beaker turned completely white. After cooling the sample to room temperature, 5 mL (feed, plasma, and urine) or 10 mL (feces) of 5% (*v/v*) nitric acid was added; the sample was allowed to stand at room temperature for 24 h to completely dissolve any remaining material. Thereafter, 5 µL (feed, plasma, and urine) or 10 µL (feces) of 1 µg/mL indium (In) was added as the internal standard solution and mixed well; each sample solution was transferred to a polycarbonate cup for ICP-MS measurement. Tall beakers and sample cups were previously soaked in 1% nitric acid for at least three days, washed with ultrapure water, and dried.

For 16S rRNA gene amplicon library construction and sequencing, DNA from fecal samples (approximately 200 mg of biomass per sample) was extracted using the ISOSPIN Fecal DNA Kit (Nippon Gene Co., Ltd., Tokyo, Japan) according to the manufacturer’s instructions. For cell lysis, three rounds (1 min each) of bead beating were performed using the FastPrep-24 instrument (MP Biomedicals, Irvine, CA, USA) at a speed of 6 m/s. DNA concentrations were measured using the Quant-iT PicoGreen dsDNA Assay Kit with a Qubit fluorometer (both from Invitrogen). Amplicon sequencing libraries were prepared by two-step tailed polymerase chain reaction (PCR) following Illumina’s “16S Metagenomic Sequencing Library Preparation” protocol. In the first round of PCR, the V4 hypervariable region of the 16S rRNA gene was amplified using primers 515F (5′-GTGYCAGCMGCCGCGGTAA-3′) [55] and 806R (5′-GGACTACNVGGGTWTCTAAT-3′) [56]; the primers contained appropriate 5′-end adapters required for indexing in the second round of PCR. The reaction mixtures (20 μL) consisted of 1× KAPA HiFi HotStart ReadyMix (F. Hoffmann-La Roche AG, Basel, Switzerland), 500 nM each of forward and reverse primers, and 5 ng of template DNA. The thermal cycling conditions were as follows: 95 °C for 3 min; followed by 18 cycles at 95 °C for 30 s, 50 °C for 30 s, and 72 °C for 30 s; and finally, 72 °C for 5 min. The PCR products were purified using the Agencourt AMPure XP system (1× volume of AMPure beads) and eluted in 50 μL of 10 mM Tris-HCl buffer (pH 8.0). The reaction mixture for the second round of PCR (30 μL) contained 1× KAPA HiFi HotStart ReadyMix, 3 μL each of i5 and i7 Nextera XT indexing oligos (Illumina, San Diego, CA, USA), and 3 μL of purified first-round PCR product. The thermal cycling conditions were as follows: 95 °C for 3 min; followed by eight cycles at 95 °C for 30 s, 55 °C for 30 s and 72 °C for 30 s; and finally, 72 °C for 5 min. Following purification using 1× AMPure beads, DNA concentrations were measured using the Quant-iT PicoGreen dsDNA Assay Kit and amplicon libraries were pooled at equimolar concentration. The pooled library was supplemented with phiX DNA (30% final concentration) and sequenced on a MiSeq instrument using V2 chemistry (2 × 251 bp reads).

### 4.4. Measuring Instruments and Measurement Parameters

GC—2010 Plus (Shimadzu Co., Kyoto, Japan) and LCMS-8060 (Shimadzu Co.) were used for targeted lipidomics. ThermoScientific Orbitrap Fusion Lumos with ThermoScientific Vanquish DGP system (Thermo Fisher Scientific Inc, Waltham, MA, USA) and ThermoScientific Orbitrap Fusion Lumos with ThermoScientific Ultimate 3000 RS (ThermoScientific) were used for untargeted metabolomics, and an inductively coupled plasma-mass spectrometer (ICP-MS; Agilent 7700/Mass Hunter, Agilent Technologies, Inc., Santa Clara, CA, USA) was used for measurements.

A FAMEWAX capillary column (30 m × 0.25 mm I.D × 0.25 μm, (Restek Corporation, Bellefonte, PA, USA ) was used for GC-FID analysis. The flow rate of the carrier gas (He) was set at 45 cm/s linear velocity. The temperatures of the injection unit and the detector were 240 °C and 250 °C, respectively. The temperature of the column oven was initiated at 140 °C, then raised to 200 °C at a rate of 11 °C/min, then increased to 225 °C at a rate of 3 °C/min, and finally increased to 240 °C at a rate of 20 °C/min, and held at this temperature for 5 min. The injection volume was 2 μL in the split injection mode. Fatty acid methyl esters were identified and quantified using a mixture of Supelco 37 component FAME Mix (Merck KGaA, Darmstadt, Germany), C22:5 (n-3)-FAME (Sigma-Aldrich), C22:5 (n-6)-FAME (Nu-Chek Prep. Inc., Elysian, MN, USA), and C22:4 (n-6)-FAME (Cayman Chemical, Ann Arbor, MI, USA) for calibration.

For untargeted lipidomics, an AcquityUPLC BEH C8 column (1.7 μm, 2.1 mm × 100 mm, Waters) was used for reversed-phase chromatography, and column temperature was 47 °C. The injection volume was 5 μL. A gradient analysis was performed with 5 mM NH_4_HCO_3_/Acetonitrile/2-propanol as the mobile phase at a flow rate of 0.35 mL/min for 55 min per analysis. Pump gradient times were as follows: 0 min (5 mM NH_4_HCO_3_/Acetonitrile/2-propanol: 75/20/5)—20 min (20/75/5)—40 min (20/5/75)—45 min (5/5/90)—50 min (5/5/90)—55 min (75/20/5). Targeted lipidomics for fatty acid-derived lipid mediators and their related metabolites was performed as described previously with some modifications [57]. Kinetex C8 column (2.6 μm, 2.1 mm × 150 mm, Phenomenex, Torrance, CA, USA) was used and 5 μL of the prepared sample was injected. Gradient analysis was performed with 0.1% formic acid in water/Acetonitrile as the mobile phase at a flow rate of 0.4 mL/min, as described previously, for urine samples with a 27 min gradient program. For plasma and feces samples, extra wash was performed with 2-propanol. The washing method is as follows; 27 min (0.1% formic acid in water/Acetonitrile/2-propanol: 5/95/0) —30 min (5/50/45)—35 min (5/50/45)—38 min (5/5/90)—42 min (5/5/90)—42.1 min (5/95/0)—44 min (5/95/0)—44.1 min (90/10/0)—48 min (90/10/0).

The trace elements (magnesium [Mg], calcium [Ca], chromium [Cr], manganese [Mn], iron [Fe], cobalt [Co], copper [Cu], zinc [Zn], and selenium [Se]) in the solutions were identified and quantitated by ICP-MS (Agilent7700/Mass Hunter, Agilent Technologies, Santa Clara, CA). Standard curves were plotted by preparing 1000 µg/mL (ppm) standard solutions of Mg, Ca, Cr, Mn, Fe, Co, Cu, Zn, and Se (Fujifilm Wako Pure Chemical Industries Ltd., Osaka, Japan) and diluting them in 5% (*v/v*) nitric acid to final metal concentrations of 0, 1, 2, 5, 10, 20, 50, 100, and 200 ng/mL (ppb). For quality control, 1 ng/mL (ppb) of a reference internal standard (indium; In) was measured along with the samples. The standard curves of each trace metal exhibited good linear regression (over r = 0.999) in the 1–200 ng/mL (ppb)concentration range. Additionally, all elements placed in the mass number table by Agilent were semi-quantified simultaneously according to the analytical method recommended by Agilent. Briefly, mass intensities of all elements were corrected and semi-quantified by those of the ICP-MS tuning solution, including five standard elements, lithium (Li), Co, yttrium (Y), cerium (Ce), and thallium (Tl), at each final concentration of 1 ng/mL (ppb).

### 4.5. Sequencing Data Processing and Analysis

Demultiplexed sequencing reads were subjected to primer trimming using Cutadapt v3.2 [58], with the following command line options: -g ^ GTGYCAGCMGNNNNNNNNN -G ^ GGACTACNVGNNNNNNNNNN --no-indels --error-rate 0.2 --discard-untrimmed --max-n 0 --minimum-length 200:200. Amplicon sequence variants (ASVs) were then inferred using DADA2 v1.16.0 [59], following additional trimming and filtering of reads using DADA2′s filterAndTrim function, specifying options truncLen = c (180, 180) and maxEE = c (3, 3). Learning of error models for forward and reverse reads (function learn Errors), denoising of forward and reverse reads (function data), merging of reads (function mergePairs), and removal of bimeras (function removeBimeraDenovo) were performed with default settings, with the exception that a minimal number of 2—108 bases from randomly selected samples was used option 2—108 (option nbases = 2 × 10^8^ and randomize = TRUE). Taxonomy of the inferred ASVs was assigned using RDP’s naïve Bayesian classifier v2.13 [60] specifying option --format fixrank.

Subsequently, a filtered ASVs table was generated by discarding ASVs lacking domain- and/or phylum-level taxonomic assignment (a bootstrap confidence score of 80%) as well as ASVs with an aberrant length (that is, shorter than 250 or longer than 255 bp based on the expected length of 253 bp for the V4 hypervariable region of the 16S rRNA gene). The filtered ASV table was 10 times randomly subsampled to 60,000 total counts per sample using vegan v2.5 (https://CRAN.R-project.org/package=vegan (accessed on 24 March 2021)) rarefy function, averaged across subsamples (arithmetic mean), and converted to relative abundances by total sum scaling. Further, duplicate measurements for each monkey were averaged prior to downstream analysis by obtaining the arithmetic mean of ASV-wise relative abundances.

To correlate ASV relative abundance with water content, we considered ASVs detected (that is, with non-zero count in the rarified ASV table) in at least a half of the samples. Spearman correlation coefficients and *p*-values were calculated using R v4.0.2 stats’s cor.test function and adjusted *p*-values generated using R stats’s p.adjust function (method = “fdr”), with a cut-off of 0.1 for significant correlations. Graphics were generated using ggplot2 v3.3.3 (https://ggplot2.tidyverse.org (accessed on 24 March 2021)) and dplyr v1.0.3 (https://CRAN.R-project.org/package=dplyr (accessed on 24 March 2021)) in R.

### 4.6. Lipid Data Analysis

Lipid species in plasma, urine, and stool samples of 20 monkeys were identified by LipidSearch4.2 using raw data files from mass spectrometry, aligned among monkeys, and then Spearman’s rank correlation coefficients were calculated for each sample based on arrays of quantitation values from 20 monkeys with all combinations of identified lipids. The search parameters of LipidSearch were set as follows: precursor ion m/z tolerance and product ion m/z tolerance were both set to 5 ppm, intensity threshold of product ion was set to 1% of the precursor ion. If peaks within 5 ppm were detected more than once within a retention time of 0.1 min, they were considered the same lipid. The m-score was set to 5 or higher, and the lipid was identified if the grade (lipid subtype and fatty acid chains [full or partial] were identified) was displayed by the LipidSearch criteria.

For data analysis, Labsolutions version 5.61 (Shimadzu Co.), GCsolution version 2.44 (Shimadzu Co.), Compound Discoverer 2.1 (Thermo Fisher Scientific Inc.), LipidSearch version 4.2 (Thermo Fisher Scientific Inc.), HMDB (https://hmdb.ca/ (accessed on 28 June 2021)), MetaboAnalyst (https://www.metaboanalyst.ca/ (accessed on 28 June 2021)), and MS Excel 2016 (Microsoft Corp., Redmond, WA, USA) were used for the calculation of Spearman’s rank correlation and *p*-values, and *p*-values for the correlation coefficient were calculated in MS Excel (Microsoft Corp.) (https://www.statology.org/p-value-correlation-excel/ (accessed on 28 June 2021)).

## Figures and Tables

**Table 1:** Stool water content and animal age. Asterisk (*) monkeys were observed to be soft stools.

Monkey	Water %	Age (Years)
1	57.93	5.8
2 *	74.50	5.1
3 *	74.07	4.2
4	65.52	4.7
5 *	77.21	5.0
6	71.46	5.1
7 *	79.45	6.3
8	72.62	5.1
9	67.80	5.6
10	69.09	6.3
11	72.98	6.2
12	75.36	5.1
13	62.24	6.3
14	57.89	5.3
15	66.60	6.1
16	64.70	4.2
17	72.67	3.9
18	67.69	4.4
19	65.50	3.8
20 *	76.92	4.0

**Table 2:** Highly correlated (>0.6) oxidized fatty acids in feces with stool water content.

Lipid Name	Correlation Coefficient	*p*-Value
13-HpODE	0.732	0.00024
9-HpODE	0.717	0.00038
12,13-EpOME	0.71	0.00045
13-HOTrE	0.659	0.0016
13-HODE	0.651	0.0019
13-KODE	0.618	0.0037

## Data Availability

All sequencing data have been deposited at NCBI’s Sequence Read Archive repository under BioProject PRJNA715597.

## Supplementary Materials

The following are available online at https://www.mdpi.com/article/10.3390/metabo11100701/s1, Table S1: Plasma metabolomics, Table S2: Urinary metabolomics, Table S3: Fecal metabolomics, Table S4: HMBD ID numbers for candidate metabolites correlated with stool water content, Table S5: Total fatty acids in plasma measured using gas chromatography, Table S6: Food lipidomics, Table S7: Fecal lipidomics, Table S8: Plasma lipidomics, Table S9: Urinary lipidomics, Table S10: Oxidized fecal fatty acids, Table S11: Oxidized plasma fatty acids, Table S12: Oxidized urinary fatty acids, Table S13: Plasma (ng/mL), fecal (ng/g of feces), and urinary (ng/mL) metallomics, Table S14: Food metallomics (ng/g), Figure S1: Scatter plots of amplicon sequence variant (ASV) abundance (*y*-axis) and water content (*x*-axis) for ASVs with a significant (adjusted *p*-value of <0.1) positive Spearman’s correlation coefficient, Figure S2: Bar chart of correlation coefficients of the association between relative abundance and water content for amplicon sequence variant (ASV) tables aggregated at the family (top panel) and genus (bottom panel) levels.

## Lipid Abbreviations

LPA (lysophosphatidic acid), PA (phosphatidic acid), LPC (lysophosphatidylcholine), PC (phosphatidylcholine), LPE (lysophosphatidylethanola-mine), PE (phosphatidylethanolamine), LPG (lysophosphatidylglycerol), PG (phosphati-dylglycerol), Hex1Cer (Hexosylceramide), Hex2Cer (Dihexosylceramide), Hex3Cer (Tri-hexosylceramide), SM (sphingomyelin), DG (diglyceride), TG (triglyceride), Pet (phospha-tidylethanol), PMe (phosphatidylmethanol).
