# Supplementary material for: Multi-Omics Analysis to Generate Hypotheses for Mild Health Problems in Monkeys
Source: Metabolites. 2021 Oct 13;11(10):701. doi: 10.3390/metabo11100701 (PMC8538200; doi:10.3390/metabo11100701)
Supplement: Supplementary file 1 [file metabolites-11-00701-s001.zip › Supplementary Figure S1.pdf]

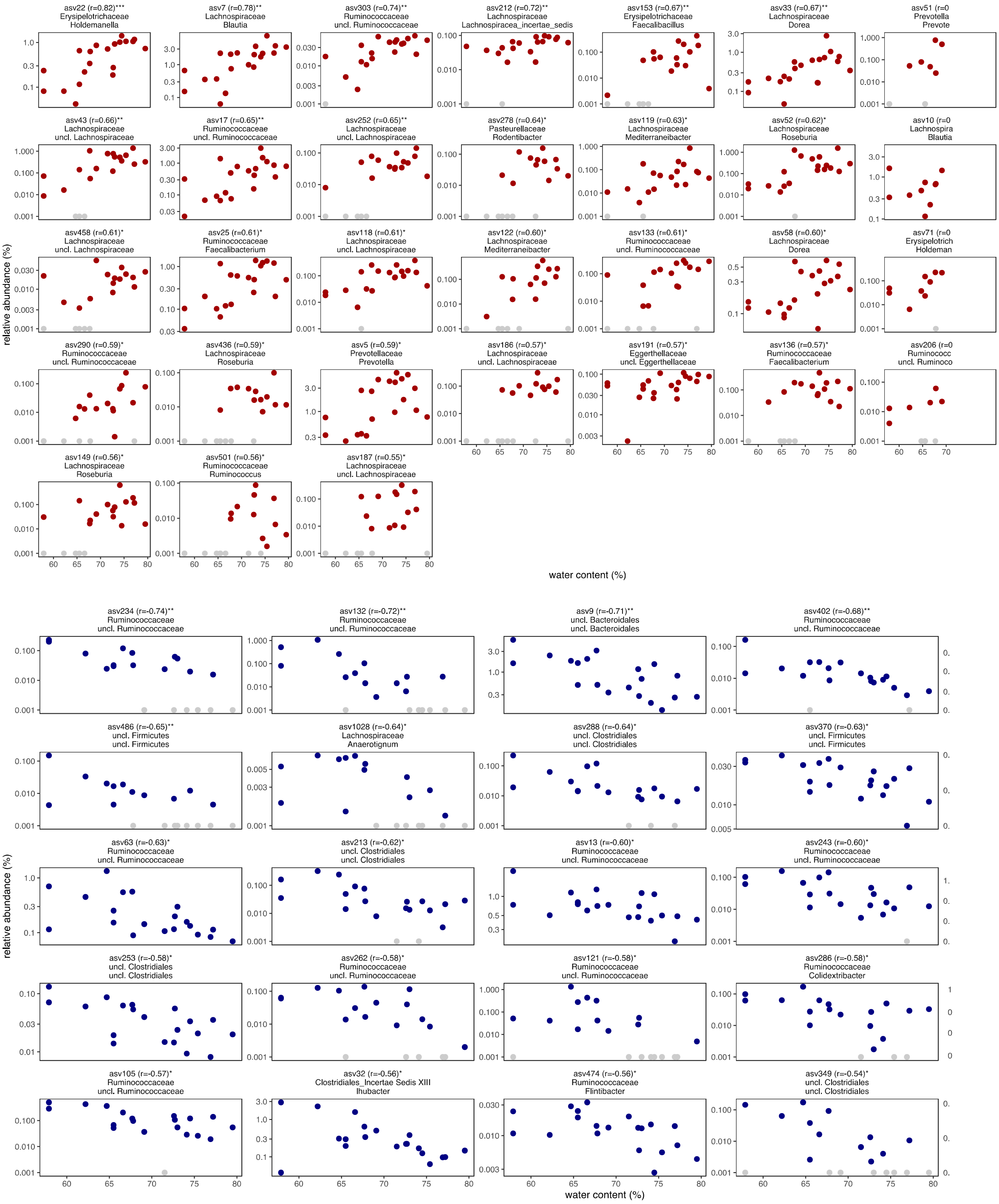

Supplementary Figure S1. Scatter plots of ASV abundance (*y*-axis) and water content (*x*-axis) for ASVs with a significant (adjusted *P*-value of <0.1) negative Spearman's correlation coefficient. Note that ASVs with zero abundance in the rarefied ASV tables are shown using light grey symbols, plotted at a pseudo-abundance of 0.001%. Each facet represents a different ASV, sorting by increasing *P*-values, as indicated in the facet labels (*** *P*-value of < 0.01, ** *P*-value of < 0.05, * *P*-value of < 0.1). Correlation coefficients (r) and family- and

genus-level taxonomic assignments are shown in the facet labels. If unclassified at the family or genus level, the lowest taxonomic rank at which the ASV could be classified was propagated to these levels and indicated as unclassified (“uncl.”)
