# Supplementary material for: Multi-Omics Analysis to Generate Hypotheses for Mild Health Problems in Monkeys
Source: Metabolites. 2021 Oct 13;11(10):701. doi: 10.3390/metabo11100701 (PMC8538200; doi:10.3390/metabo11100701)
Supplement: Supplementary file 1 [file metabolites-11-00701-s001.zip › Supplementary Figure S2.pdf]

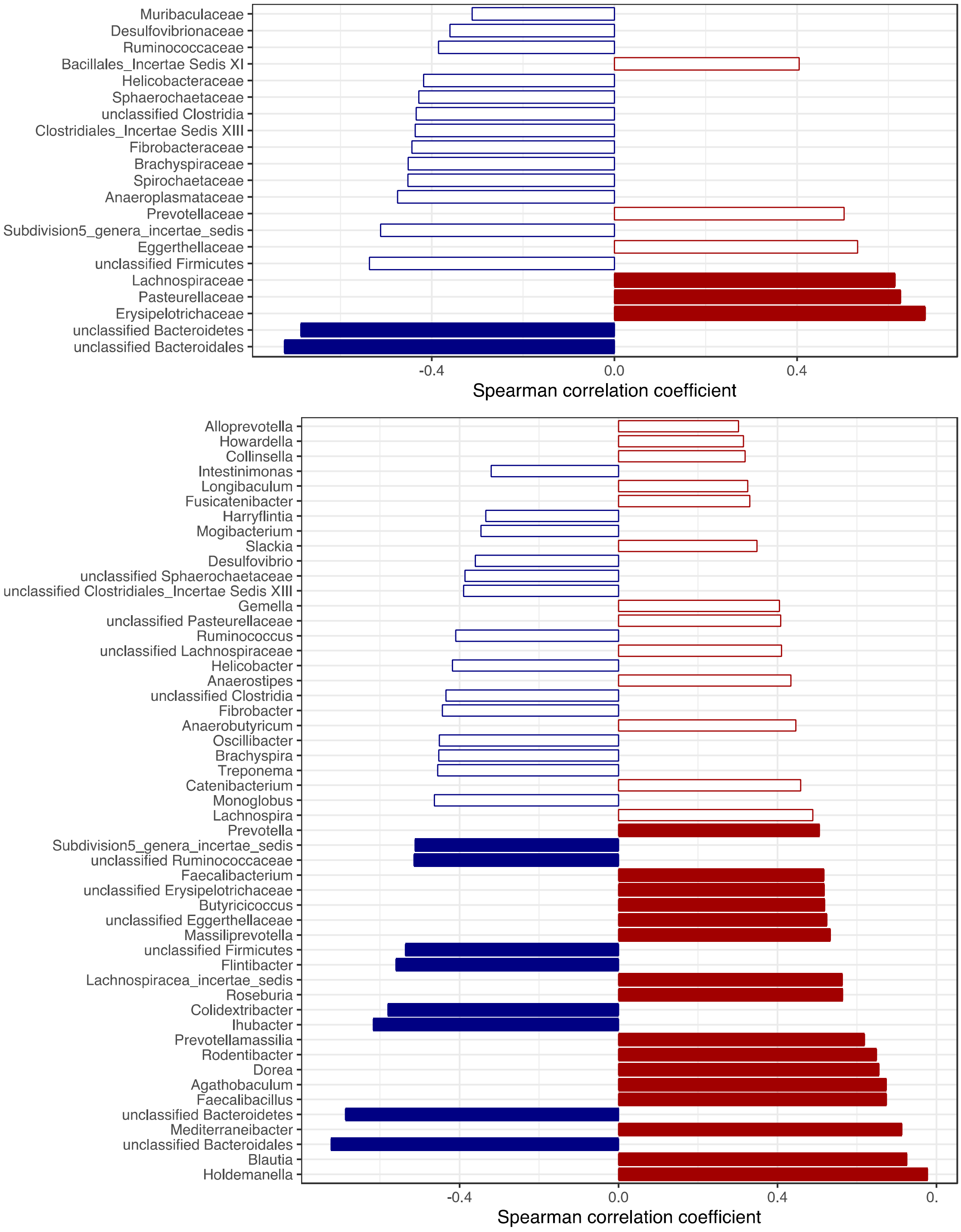

Supplementary Figure S2. Bar chart of correlation coefficients of the association between relative abundance and water content for amplicon sequence variant (ASV) tables aggregated at the family (top panel) and genus (bottom panel) levels. Only taxa (that is, families or genera) present in at least half of the samples were considered, and only taxa with a correlation coefficient of >0.3 or -0.3 are shown. Bars are colored based on the sign of the

correlation coefficient and sorted by increasing absolute value from top to bottom. Filled bars represent taxa with a significant correlation to water content (adjusted *P*-value of <0.1).
