# Supplementary material for: Multi-Omics Analysis to Generate Hypotheses for Mild Health Problems in Monkeys
Source: Metabolites. 2021 Oct 13;11(10):701. doi: 10.3390/metabo11100701 (PMC8538200; doi:10.3390/metabo11100701)
Supplement: Supplementary file 1 [file metabolites-11-00701-s001.zip › Suppl_Table_Figure_Legends.pdf]

Multi-Omics Analysis to Generate Hypotheses for Mild Health Problems in Monkeys

Supplementary materials

Table S1: Plasma metabolomics. Numbers for individual monkeys and lipids represent mass spectrometric intensity.

Table S2: Urinary metabolomics. Numbers for individual monkeys and lipids represent mass spectrometric intensity.

Table S3: Fecal metabolomics. Numbers for individual monkeys and lipids represent mass spectrometric intensity.

Table S4: HMBD ID numbers for candidate metabolites correlated with stool water content.

Table S5: Total fatty acids in plasma measured using gas chromatography. Unit is g/ml.

Table S6: Food lipidomics. Numbers for individual monkeys and lipids represent mass spectrometric intensity.

Table S7: Fecal lipidomics. Numbers for individual monkeys and lipids represent mass spectrometric intensity.

Table S8: Plasma lipidomics. Numbers for individual monkeys and lipids represent mass spectrometric intensity.

Table S9: Urinary lipidomics. Numbers for individual monkeys and lipids represent mass spectrometric intensity.

Table S10: Oxidized fecal fatty acids. Numbers for individual monkeys and lipids represent mass spectrometric intensity.

Table S11: Oxidized plasma fatty acids. Numbers for individual monkeys and lipids represent mass spectrometric intensity.

Table S12: Oxidized urinary fatty acids. Numbers for individual monkeys and lipids represent

mass spectrometric intensity.

Table S13: Plasma (ng/mL), fecal (ng/g of feces), and urinary (ng/mL) metallomics

Table S14: Food metallomics (ng/g)

Figure S1: Scatter plots of amplicon sequence variant (ASV) abundance (y-axis) and water content (x-axis) for ASVs with a significant (adjusted P-value of <0.1) positive Spearman's correlation coefficient. Note that ASVs with zero abundance in the rarefied ASV tables are shown using light grey symbols, plotted at a pseudo-abundance of 0.001%. Each facet represents a different ASV, sorting by increasing P-values, as indicated in the facet labels (*** P-value of < 0.01, ** P-value of < 0.05, * P-value of < 0.1). Correlation coefficients (r) and family- and genus-level taxonomic assignments are shown in the facet labels. If unclassified at the family or genus level, the lowest taxonomic rank at which the ASV could be classified was propagated to these levels and indicated as unclassified ("uncl.").

Figure S2: Bar chart of correlation coefficients of the association between relative abundance and water content for amplicon sequence variant (ASV) tables aggregated at the family (top panel) and genus (bottom panel) levels. Only taxa (that is, families or genera) present in at least half of the samples were considered, and only taxa with a correlation coefficient of >0.3 or -0.3 are shown. Bars are colored based on the sign of the correlation coefficient and sorted by increasing absolute value from top to bottom. Filled bars represent taxa with a significant correlation to water content (adjusted P-value of <0.1).
