# Supplementary figures and images for: Multi-Omics Analysis to Generate Hypotheses for Mild Health Problems in Monkeys
Source: Metabolites. 2021 Oct 13;11(10):701. doi: 10.3390/metabo11100701 (PMC8538200; doi:10.3390/metabo11100701)

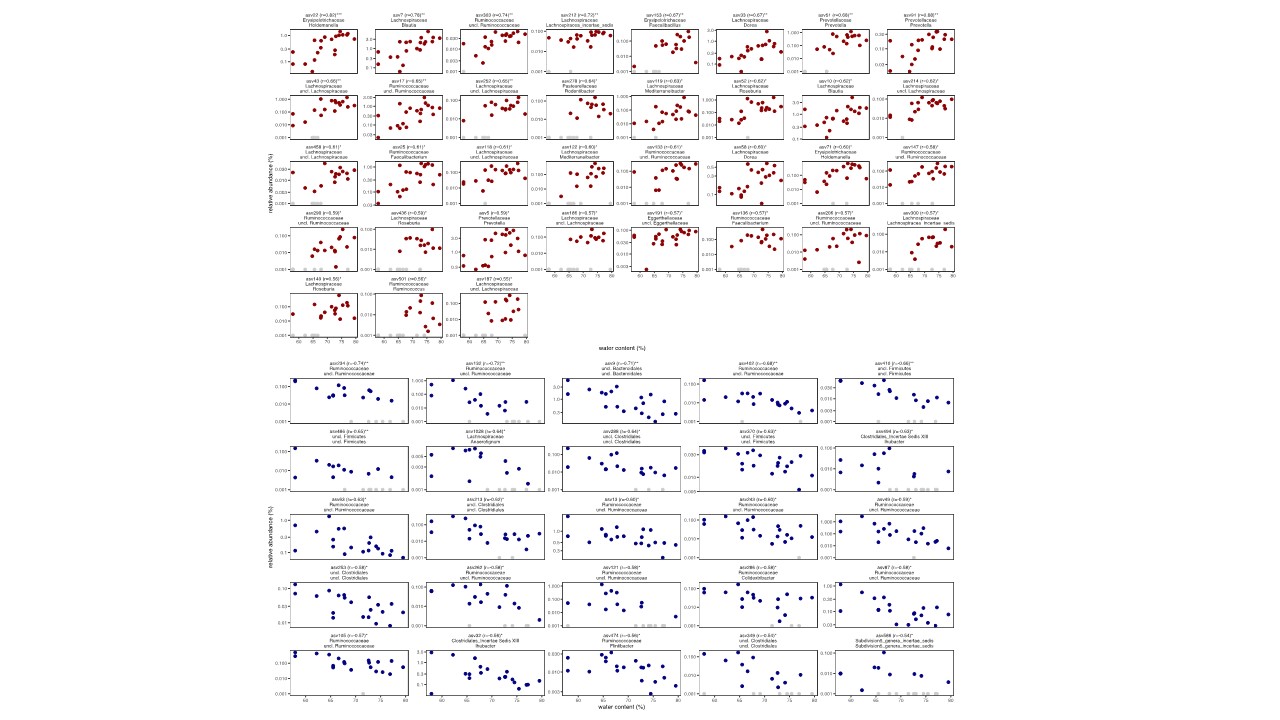

Supplement: Supplementary file 1 [file metabolites-11-00701-s001.zip › Suppl_Figure_S1.JPG]

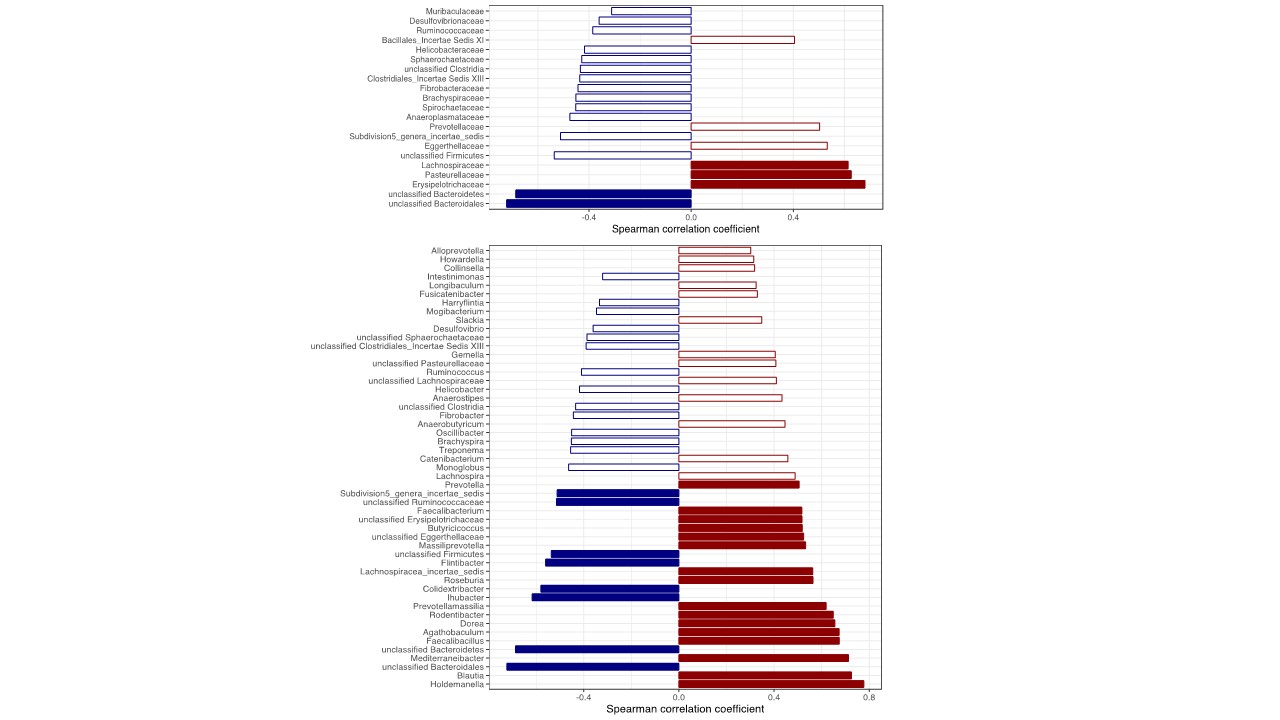

Supplement: Supplementary file 1 [file metabolites-11-00701-s001.zip › Suppl_Figure_S2.jpg]
